# Immunogenicity and Safety Following 1 Dose of AS01_E_-Adjuvanted Respiratory Syncytial Virus Prefusion F Protein Vaccine in Older Adults: A Phase 3 Trial

**DOI:** 10.1093/infdis/jiad546

**Published:** 2023-12-14

**Authors:** Tino F Schwarz, Shinn-Jang Hwang, Pedro Ylisastigui, Chiu-Shong Liu, Kenji Takazawa, Makoto Yono, John E Ervin, Charles P Andrews, Charles Fogarty, Tamara Eckermann, Delphine Collete, Magali de Heusch, Nathalie De Schrevel, Bruno Salaun, Axel Lambert, Céline Maréchal, Aurélie Olivier, Phoebe Nakanwagi, Marc Lievens, Veronica Hulstrøm

**Affiliations:** Institute of Laboratory Medicine and Vaccination Centre, Klinikum Würzburg Mitte, Germany; En Chu Kong Hospital, New Taipei City, and Taipei Veterans General Hospital and School of Medicine, National Yang Ming Chiao Tung University, Taipei, Taiwan; Alliance for Multispecialty Research, Fort Myers, Florida; China Medical University and China Medical University Hospital, Taichung, Taiwan; Medical Corporation Shinanokai, Shinanozaka Clinic, Tokyo, Japan; Nishi-Kumamoto Hospital, Kumamoto, Japan; Alliance for Multispecialty Research, Kansas City, Missouri; IMA Research San Antonio, Texas; Spartanburg Medical Research, South Carolina; Praxis Dr med Irmgard Maier-Bosse, Munich, Germany; GSK, Rixensart, Belgium; GSK, Wavre, Belgium; GSK, Rixensart, Belgium; GSK, Rixensart, Belgium; GSK, Wavre, Belgium; GSK, Wavre, Belgium; GSK, Wavre, Belgium; GSK, Wavre, Belgium; GSK, Wavre, Belgium; GSK, Wavre, Belgium

**Keywords:** immunogenicity, older adults, phase 3 study, respiratory syncytial virus, safety

## Abstract

**Background:**

The recently approved AS01_E_-adjuvanted respiratory syncytial virus (RSV) prefusion F protein–based vaccine for older adults (RSVPreF3 OA) demonstrated high efficacy against RSV-related disease in ≥60-year-olds.

**Methods:**

This ongoing phase 3 study in ≥60-year-olds evaluates immune persistence until 3 years after RSVPreF3 OA vaccination. Here, we describe interim results on humoral and cell-mediated immunogenicity, reactogenicity, and safety until 1 year post–dose 1.

**Results:**

In total, 1653 participants were vaccinated. One month post–dose 1, neutralization titers increased 10.5-fold (RSV-A) and 7.8-fold (RSV-B) vs pre–dose 1. Titers then declined to levels 4.4-fold (RSV-A) and 3.5-fold (RSV-B) above pre–dose 1 at month 6 and remained 3.1-fold (RSV-A) and 2.3-fold (RSV-B) above pre–dose 1 levels after 1 year. RSVPreF3-binding immunoglobulin G levels and CD4+ T-cell frequencies showed similar kinetics. Solicited administration-site and systemic adverse events (mostly mild to moderate and transient) were reported by 62.2% and 49.5% of participants. Serious adverse events were reported by 3.9% of participants within 6 months post–dose 1; 1 case was considered vaccine related.

**Conclusions:**

One RSVPreF3 OA dose elicited cell-mediated and RSV-A– and RSV-B–specific humoral immune responses that declined over time but remained above pre–dose 1 levels for at least 1 year. The vaccine was well tolerated with an acceptable safety profile.

***Clinical Trials Registration.*** NCT04732871 (ClinicalTrials.gov).

## Teaser

One dose of prefusion F protein–based vaccine for older adults administered to ≥60-year-olds induced humoral and cell-mediated immune responses that remained above pre–dose 1 levels for at least 1 year. The vaccine was well tolerated with an acceptable safety profile.

Respiratory syncytial virus (RSV) is a common contagious virus that causes acute respiratory illness. Most RSV infections manifest as a mild illness with cold-like symptoms, but RSV can also cause more severe lower respiratory tract diseases, such as bronchitis, bronchiolitis, and pneumonia, which may require hospitalization [1]. Although RSV is best known as a cause of respiratory morbidity and mortality in young children [2], it is increasingly being recognized as an important pathogen in adults [2, 3]. Especially older adults and those with chronic medical conditions—particularly immunocompromising and chronic cardiopulmonary diseases—are at higher risk of developing severe RSV-related disease [4, 5]. In 2019, an estimated 5.2 million adults aged ≥60 years in high-income countries got infected with RSV, of whom 470 000 were hospitalized and 33 000 died due to an RSV infection [6].

RSV circulation typically peaks in the winter months in temperate climates, while in tropical areas, infections can occur year-round but primarily manifest during rainy seasons [1, 7]. Two antigenically distinct RSV groups exist, RSV-A and RSV-B, whose predominance typically alternates with each epidemic season, although regional variation exists [3].

The immunity built up after a natural RSV infection is incomplete, and its duration is limited, often not even lasting until the next epidemic season [8]. Therefore, reinfections can occur throughout an individual's lifetime. Serum neutralization titers appear to be correlated with protection against reinfection, although they usually drop soon following an RSV infection [9–13]. Little is known about the role of virus-induced cell-mediated immune responses in the protection against RSV reinfection, but it is thought that RSV-specific T-cell responses mostly play a role in viral clearance and controlling disease severity [14–16]. Age-related reductions in T-cell responses due to immunosenescence likely contribute to the susceptibility of older adults to severe RSV illness [15].

The AS01_E_-adjuvanted prefusion F protein–based vaccine for older adults (RSVPreF3 OA, Arexvy; GSK) was approved by the US Food and Drug Administration in May 2023. The vaccine contains a recombinant subunit RSV F protein antigen (RSVPreF3) based on the RSV-A2 strain [17, 18]. The RSV F protein is a surface glycoprotein that is highly conserved across RSV-A and RSV-B and their variants, and when stabilized in its prefusion conformation, it exposes epitopes that are targeted by the most potent neutralizing antibodies [19, 20].

In the placebo-controlled Adult Respiratory Syncytial Virus phase 3 trial (AReSVi-006) among adults aged ≥60 years, a single dose of RSVPreF3 OA was well tolerated and demonstrated high efficacy against RSV-related disease during 2 RSV seasons [21, 22]. The trial is ongoing and will continue monitoring the vaccine efficacy up to 3 years postvaccination to shed light on the duration of protection of RSVPreF3 OA over longer periods.

Alongside the ongoing efficacy trial, the present ongoing phase 3 trial evaluates the persistence of the humoral and cell-mediated immune response up to 3 years following a single dose of the RSVPreF3 OA vaccine and assesses different revaccination schedules in adults aged ≥60 years and the related reactogenicity and safety profiles. Here, we describe the immunogenicity, reactogenicity, and safety results of an interim analysis based on data collected up to 1 year after a single dose of the vaccine.

## METHODS

### Study Design and Conduct

This open-label multicountry study is an ongoing phase 3 trial with 3 parallel groups of participants (randomized 3:1:1) who received the following:

• A first RSVPreF3 OA dose on day 1, followed by revaccination doses 1 year and 2 years post–dose 1

• A first RSVPreF3 OA dose on day 1 and a revaccination dose 2 years post–dose 1

• A single dose of RSVPreF3 OA on day 1

Participants will be followed up until 3 years post–dose 1. Since interventions and procedures were the same for all 3 study groups until 1 year post–dose 1, the results from the 3 groups were pooled in the current analyses.

The study is carried out at 46 centers in 5 countries: Finland (10), Germany (8), Japan (3), Taiwan (7), and the United States (18). The study was registered at ClinicalTrials.gov (NCT04732871) and is conducted according to the Declaration of Helsinki, the international ethical guidelines of the Council for International Organizations of Medical Sciences, the good clinical practice guidelines of the International Conference on Harmonization, and applicable regulatory requirements. The study documents were approved by national, regional, or study center independent ethics committees or institutional review boards. Participants provided written or witnessed informed consent prior to performance of any study-specific procedures. A protocol summary is available at https://www.gsk-studyregister.com/en/trial-details/?id=212496.

### Study Participants

Adults aged ≥60 years were screened and enrolled if the investigator judged that they would be able to comply with the protocol. Individuals with chronic medical conditions, with or without specific treatment, were eligible if the investigator considered them medically stable. Individuals who had previously received any RSV candidate vaccine or who had received or planned to receive other vaccines within 30 days of study vaccination were excluded—except inactivated, split-virion, or subunit influenza vaccines, for which the time window was shortened to 14 days, and COVID-19 vaccines, which were given according to local guidelines. Inclusion and exclusion criteria are detailed in the supplementary methods.

Participants were enrolled in 3 age categories reflecting the distribution in the general population, with approximately 40% aged 60 to 69 years, 30% 70 to 79 years, and 10% ≥80 years. The remaining 20% were distributed freely across the 3 age categories.

### Study Intervention, Randomization, and Blinding

One 0.5-mL dose of the RSVPreF3 OA vaccine consisted of (1) 120 µg of RSVPreF3 antigen derived from the RSV-A2 strain and (2) the liposome-based AS01_E_ adjuvant system, containing 25 µg of 3-O-desacyl-4′-monophosphoryl lipid A and 25 µg of *Quillaja saponaria* Molina, fraction 21. The vaccine was administered by intramuscular injection into the deltoid muscle of the nondominant arm.

Participants were randomly assigned 3:1:1 to the 3 study groups via the Source Data Base for Internet Randomization system with minimization, accounting for age, sex, center, setting (general community or long-term care facility), and cell-mediated immunogenicity (CMI) subset. Humoral immunogenicity and CMI were evaluated in subsets of participants with predefined target sample sizes, which were generated per the Source Data Base for Internet Randomization system (Supplementary Methods).

This is an open-label study, in which only the laboratory in charge of sample testing was blinded to the intervention assignment.

### Study Objectives

The primary objective was to evaluate the humoral immune response until 1 year after a single dose of the RSVPreF3 OA vaccine by measuring RSV-A and RSV-B neutralization titers. The main secondary immunogenicity objectives were to further evaluate the humoral immune response in terms of RSVPreF3-binding immunoglobulin G (IgG) concentrations and to characterize the CMI response in terms of the frequency of RSVPreF3-specific polypositive CD4+ and/or CD8+ T cells. Reactogenicity and safety were also evaluated as secondary objectives (supplementary methods).

### Immunogenicity Evaluation

Blood samples for humoral immunogenicity and CMI analyses were collected from participants in the humoral immunogenicity and CMI subsets at day 1, day 31, month 6, and month 12. RVS-A and RSV-B neutralization titers were assessed by using in-house neutralization assays that make use of 2 RSV strains: Long (VR-26; RSV-A) and 18537 (VR-1580; RSV-B). RSVPreF3-binding IgG concentrations were assessed with an in-house enzyme-linked immunosorbent assay [17]. Frequencies of RSVPreF3-specific polypositive CD4+ and/or CD8+ T cells expressing at least 2 activation markers—including at least 1 cytokine among CD40 ligand, 4-1BB, interleukin 2, tumor necrosis factor α, interferon γ, interleukin 13, and interleukin 17—were measured via an intracellular cytokine staining assay of peripheral blood mononuclear cell samples, with a lower limit of detection of 310.0 and a lower limit of quantification of 590.0 polypositive CD4+ and/or CD8+ T cells per 1 million cells [17].

### Reactogenicity and Safety Evaluation

Using paper diary cards, participants recorded solicited administration-site and systemic adverse events (AEs) during 4 days following vaccine administration and unsolicited AEs during 30 days following vaccine administration. The investigators transcribed the information of the events into the electronic case report forms and determined the relationship between the vaccine and the occurrence of the unsolicited AEs. The intensity of AEs was graded from mild (grade 1) to severe (grade 3; supplementary methods).

Serious AEs (SAEs) and potential immune-mediated diseases were recorded by the investigator from the time of vaccine administration up to month 6 and were followed up until the event was resolved, stabilized, or otherwise explained or until the participant was lost to follow-up.

SAEs and potential immune-mediated diseases considered related to study vaccination by the investigator, fatal SAEs, and AEs/SAEs leading to withdrawal from the study are recorded until study end; those recorded until the current data lock point (approximately 12 months post–dose 1) are reported in this article.

### Statistical Analyses

We planned to enroll approximately 1650 participants in this trial. Safety and demographic characteristics were assessed on the exposed set: all participants who received 1 dose. Humoral immunogenicity and CMI were assessed on the per-protocol sets—all participants who received at least 1 dose, complied with eligibility criteria and study procedures, and had the postvaccination immunogenicity results available—at the following time points: day 1, day 31, month 6, and month 12.

Demographic variables were summarized with descriptive statistics (mean, median, SD, range) for continuous variables or frequencies (number, percentage) for categorical variables. RSV-A and RSV-B geometric mean neutralization titers and RSVPreF3-binding IgG geometric mean concentrations were calculated with 95% CIs. The mean geometric increases—specifically, the geometric means of ratios of neutralization titers or antibody concentrations for each postvaccination time point over prevaccination—were calculated with 95% CIs. The percentage of participants with neutralization titers/IgG concentrations equal to or above the assay cutoffs were calculated with exact 95% CIs. CMI was evaluated by descriptive statistics at each time point (median, minimum, maximum, 25th and 75th percentiles). The immunogenicity analyses were also done by age category (60–69, 70–79, ≥80 years), sex, and region (North America, Europe, Asia). Safety data were evaluated by descriptive statistics with frequencies (number and percentage with exact 95% CIs). Statistical analyses were performed with the SAS Life Science Analytics Framework.

## RESULTS

### Study Population

**Figure 1.**
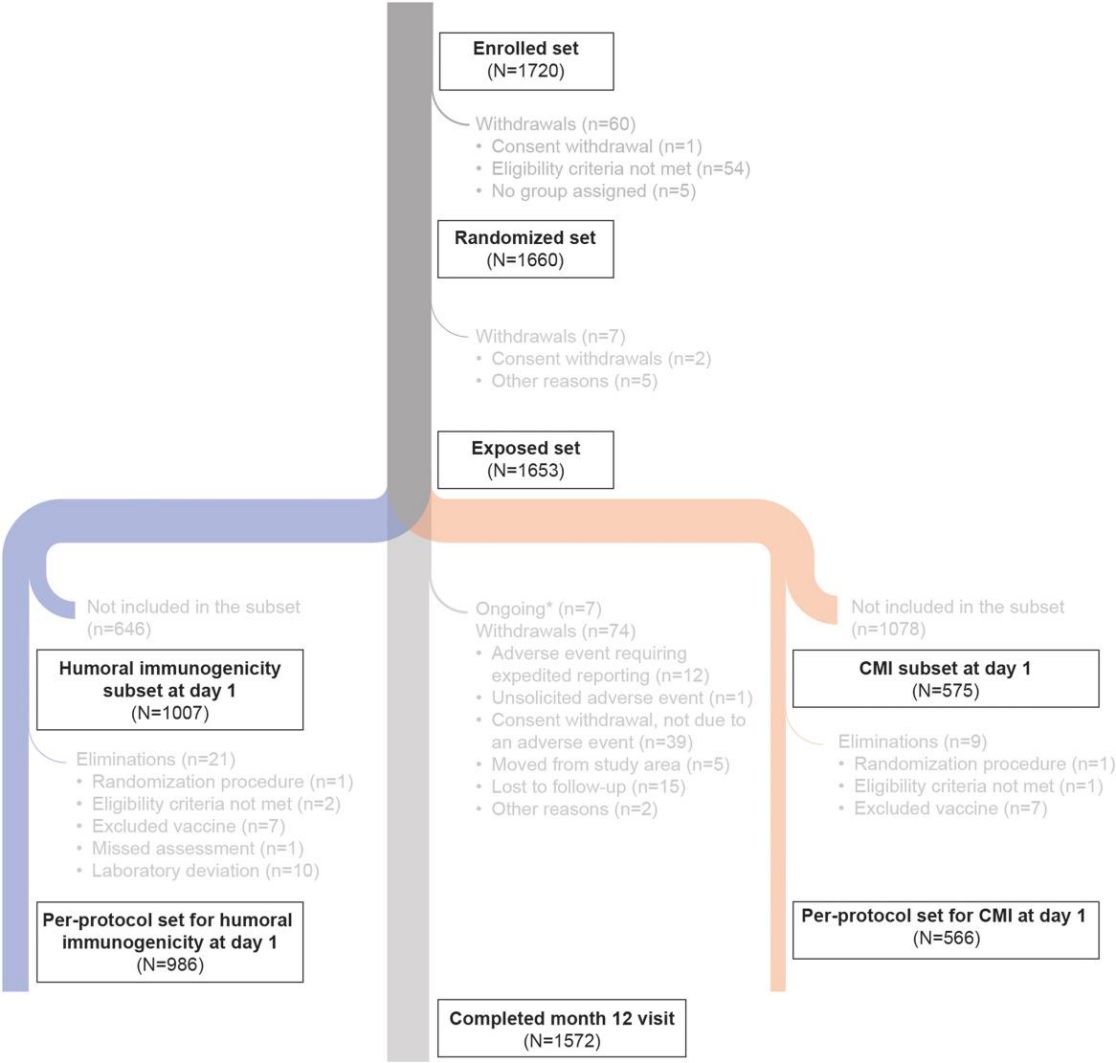
Flow of participants. *Number of participants who were still participating in the study at the data lock point but did not yet complete their month 12 visit. CMI, cell-mediated immunogenicity.

A total of 1720 participants were screened and enrolled between 15 February 2021 and 10 May 2021. Of these, 60 participants were withdrawn before randomization, mainly because eligibility criteria were not met. In total, 1660 were randomized, and 1653 received 1 RSVPreF3 OA dose at day 1 and were included in the exposed set. Out of the 1007 participants randomized to the humoral immunogenicity subset, 986 were included in the per-protocol set for humoral immunogenicity, and out of the 575 participants randomized to the CMI subset, 566 were included in the per-protocol set for CMI. A total of 74 participants (4.5%) were withdrawn from the study at different time points up to month 12, mainly due to consent withdrawal, not due to an AE/SAE (2.4%; Figure 1).

Of all participants in the exposed set, 54.7% were female. The mean ± SD age of the participants on day 1 was 70.0 ± 6.6 years; 49.6% were 60 to 69 years, 37.6% were 70 to 79 years, and 12.8% were ≥80 years (Table 1).

**Table 1.** Demographics and Baseline Characteristics of Study Participants: Exposed Set

Characteristic	Value or No. (%)
No.	1653
Age, y	
Mean ± SD	70.0 ± 6.6
60–69	820 (49.6)
70–79	621 (37.6)
≥80	212 (12.8)
Female sex	904 (54.7)
Ethnicity: not Hispanic or Latino	1621 (98.1)
Race	
American Indian or Alaska native	2 (0.1)
Asian	496 (30.0)
Black or African American	33 (2.0)
White	1121 (67.8)
Other	1 (0.1)
Geographic region	
Asia	493 (29.8)
Europe	727 (44.0)
North America	433 (26.2)
Type of residence	
Community dwelling	1652 (99.9)
Long-term care facility	1 (0.1)
Smoking status	
Never smoked	982 (59.4)
Current smoker	176 (10.6)
Former smoker	495 (29.9)

### Immunogenicity

#### Humoral Immunogenicity

**Figure 2.**
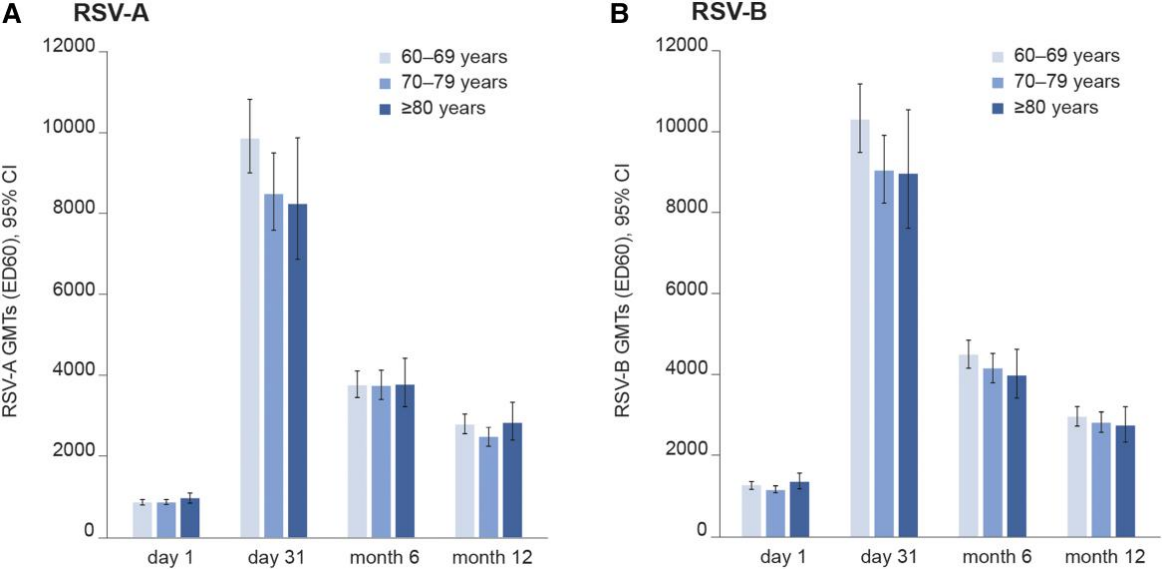
Neutralization titers by age group: *A*, RSV-A; *B*, RSV-B. Per-protocol set for humoral immunogenicity. Error bars represent 95% CIs. ED60, estimated dilution 60; GMT, geometric mean neutralization titer; RSV, respiratory syncytial virus.

At baseline (day 1), all tested participants had detectable RSV-A and RSV-B neutralization titers due to previous exposure to RSV. Neutralization titers increased 10.5-fold for RSV-A and 7.8-fold for RSV-B between prevaccination and 1 month postvaccination (day 31). After this initial sharp increase, titers declined to levels that were 4.4-fold (RSV-A) and 3.5-fold (RSV-B) higher than pre–dose 1 levels at month 6, but they remained above prevaccination levels up to month 12: 3.1-fold for RSV-A and 2.3-fold for RSV-B (Table 2). The magnitude and kinetics of RSV-A and RSV-B neutralization titers were similar across all age groups (Figure 2, Supplementary Table 1). No sex differences (data not shown) or geographic differences (Supplementary Table 2) in immune responses were observed.

**Table 2.** RSV-A and RSV-B Neutralization Titers and RSVPreF3-Binding IgG Concentrations: Per-Protocol Set for Humoral Immunogenicity

Time Point	No.	GMT/GMC (95% CI)	No.	MGI (95% CI)
RSV-A neutralization titer^a^				
Day 1	985	863.4 (819.7–909.4)	…	…
Day 31	937	9096.5 (8509.0–9724.5)	936	10.5 (9.9–11.2)
Month 6	924	3749.0 (3532.0–3979.5)	923	4.4 (4.2–4.6)
Month 12	870	2667.2 (2505.5–2839.4)	869	3.1 (3.0–3.3)
RSV-B neutralization titer^a^				
Day 1	986	1235.0 (1171.2–1302.1)	…	…
Day 31	937	9627.0 (9084.7–10 201.6)	937	7.8 (7.3–8.3)
Month 6	924	4295.7 (4069.5–4534.4)	924	3.5 (3.4–3.7)
Month 12	870	2886.1 (2724.2–3057.7)	870	2.3 (2.2–2.5)
RSVPreF3 IgG concentration^b^				
Day 1	985	7486.9 (7194.9–7790.7)	…	…
Day 31	937	91 123.5 (87 326.7–95 085.3)	936	12.2 (11.6–12.8)
Month 6	924	35 162.8 (33 679.8–36 711.2)	923	4.7 (4.5–5.0)
Month 12	870	26 161.1 (25 098.1–27 269.1)	870	3.5 (3.4–3.6)

Abbreviations: ED60, estimated dilution 60; EU, enzyme-linked immunosorbent assay units; GMC, geometric mean concentration; GMT, geometric mean neutralization titer; IgG, immunoglobulin G; MGI, mean geometric increase at given time point over baseline (day 1); RSV, respiratory syncytial virus; RSVPreF3, RSV prefusion F protein.

^a^GMT (95% CI), ED60.

^b^GMC (95% CI), EU/mL.

Similar to what was observed for RSV-A and RSV-B neutralization titers, vaccination induced an RSVPreF3-binding IgG response, with antibody concentrations increasing 12.2-fold at day 31, declining to 4.7-fold at month 6, and remaining 3.5-fold higher at month 12 when compared with prevaccination levels (Table 2).

#### Cell-Mediated Immunogenicity

**Figure 3.**
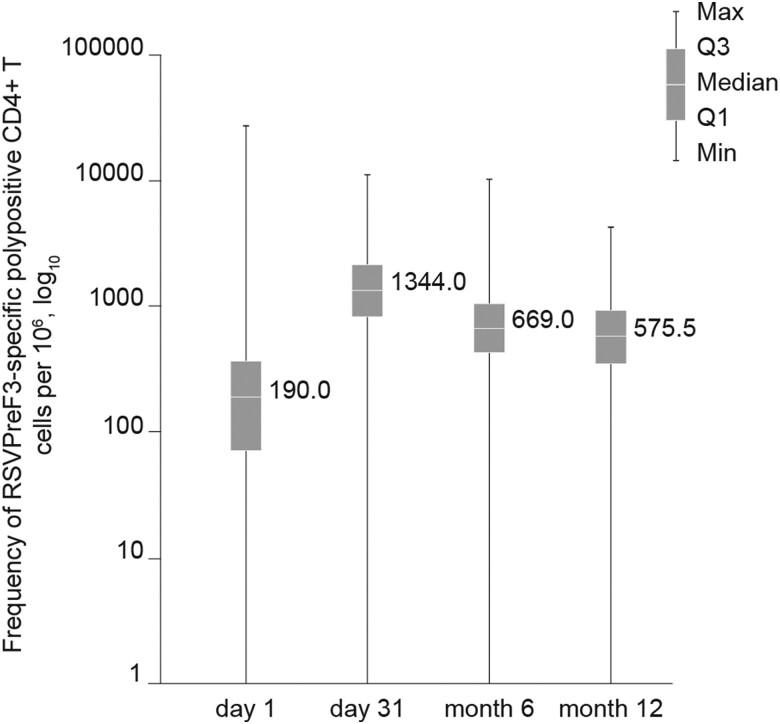
Frequency of RSVPreF3-specific polypositive CD4+ T cells expressing at least 2 activation markers, including at least 1 cytokine among CD40L, 4-1BB, IL-2, TNF-α, IFN-γ, IL-13, and IL-17 overall. Per-protocol set for cell-mediated immunogenicity. The number of samples ranged between 408 and 471 depending on the time point. CD4/CD40L, cluster of differentiation 4/40 ligand; IFN-γ, interferon gamma; IL, interleukin; RSVPreF3, respiratory syncytial virus prefusion F protein; TNF-α, tumor necrosis factor alpha.

At baseline, the median frequency of RSVPreF3-specific polypositive CD4+ T cells (per million CD4+ T cells) expressing at least 2 activation markers—including at least 1 cytokine among CD40 ligand, 4-1BB, interleukin 2, tumor necrosis factor α, interferon γ, interleukin 13, and interleukin 17—was 190.0, which was below the lower limit of detection (310.0). This value increased to 1344.0 at day 31, then declined to 669.0 at month 6, but remained well above the baseline values at 575.5 at month 12, just below the quantification limit of 590.0 and above the lower limit of detection (Figure 3). Again, the magnitude and kinetics of the CD4+ T-cell response were similar across all age groups (Supplementary Table 3). No RSVPreF3-specific CD8+ T-cell response was observed (data not shown).

### Reactogenicity and Safety

Within 4 days post–dose 1, 62.2% of participants reported solicited administration-site AEs, with pain being the most frequent event (60.5% of participants); 1.5% of participants indicated at least 1 grade 3 solicited administration-site AE. Nearly half of participants (49.5%) reported solicited systemic AEs: the most frequent events were myalgia (33.5%) and fatigue (31.4%), and 2.9% indicated a grade 3 solicited systemic AE (Table 3). The median duration of administration-site and systemic AEs was 2 days (data not shown).

**Table 3.** Solicited and Unsolicited Adverse Events, Serious Adverse Events, and Potential Immune-Mediated Diseases: Exposed Set

Event	No.	% (95% CI)
Solicited AEs (up to 4 d post–dose 1)		
Administration-site (N = 1645)		
Any grade	1024	62.2 (59.8–64.6)
Grade 3	25	1.5 (1.0–2.2)
Pain	996	60.5 (58.1–62.9)
Erythema	159	9.7 (8.3–11.2)
Swelling	124	7.5 (6.3–8.9)
Systemic (N = 1646)		
Any grade	815	49.5 (47.1–52.0)
Grade 3	48	2.9 (2.2–3.8)
Fever (≥38 °C)	25	1.5 (1.0–2.2)
Headache	336	20.4 (18.5–22.4)
Fatigue	517	31.4 (29.2–33.7)
Myalgia	551	33.5 (31.2–35.8)
Arthralgia	255	15.5 (13.8–17.3)
Unsolicited AEs (up to 30 d post–dose 1; N = 1653)		
Any grade	212	12.8 (11.3–14.5)
Grade 3	20	1.2 (.7–1.9)
Considered related to vaccine	59	3.6 (2.7–4.6)
Serious AEs (N = 1653)		
Any SAE (up to 6 mo)	65	3.9 (3.0–5.0)
Any SAE considered related to vaccine (up to DLP)^a^	1	0.1 (.0–.3)
Fatal SAE (up to DLP)	7	0.4 (.2–.9)
Fatal SAE considered related to vaccine (up to DLP)	0	…
pIMD (N = 1653)		
Any pIMD (up to 6 mo)	7	0.4 (.2–.9)
pIMD considered related to vaccine (up to DLP)^a^	1	0.1 (.0–.3)

Abbreviations: AE, adverse event; DLP, data lock point; pIMD, potential immune-mediated disease; SAE, serious adverse event.

^a^Case of Guillain-Barré syndrome: fully resolved.

Within 30 days post–dose 1, 12.8% of participants reported at least 1 unsolicited AE; 1.2% had at least 1 grade 3 unsolicited AE; and 3.6% had at least 1 unsolicited AE considered by the investigator as related to the vaccine (Table 3). The most frequent unsolicited events were headache (1.1%), arthralgia (0.7%), cough (0.6%), and injection-site pruritus (0.6%). The most frequent vaccine-related unsolicited AEs were injection-site pruritus (0.6%) and chills (0.5%).

Up to month 6, 3.9% of participants reported at least 1 SAE, and 0.4% indicated at least 1 potential immune-mediated disease (Table 3). Atrial fibrillation was the most common SAE (0.3% of participants; Supplementary Table 4). One SAE (Guillain-Barré syndrome) was assessed as related to the vaccine by the investigator. The symptoms of this case appeared 9 days after vaccination, and the participant was hospitalized. The participant recovered after 6 months and could resume daily life activities without disability. During hospitalization, elevated cerebrospinal fluid protein levels and positive serum ganglioside-monosialic acid IgG were measured. No neurologist was consulted, and no electrophysiologic studies were performed. Despite a temporal relationship of this case with the RSVPreF3 OA vaccine, the diagnosis was not conclusive for Guillain-Barré syndrome based on the Brighton Collaboration Working Group case definition [23], and alternative diagnoses could be considered. No vaccine-related SAEs occurred between month 6 and month 12. Up to the data lock point, 7 (0.4%) participants died. None of the fatal SAEs were considered by the investigator to be causally related to the vaccine.

## DISCUSSION

The present data of this ongoing phase 3 study indicate that 1 dose of the RSVPreF3 OA vaccine elicits RSV-specific humoral and cell-mediated immune responses that persist at least up to 1 year after vaccination in adults aged ≥60 years.

Humoral RSV-A– and RSV-B–specific responses and cell-mediated immune responses showed similar longitudinal profiles, with a sharp increase at 1 month post–dose 1, followed by a decline that was more pronounced between day 31 and month 6 than after month 6. Antibody levels and CD4+ T-cell frequencies remained well above baseline up to 1 year postvaccination. These observations are consistent with initial immunogenicity results after 2 doses of the vaccine administered 2 months apart in a phase 1/2 study [17] and provide additional evidence in favor of the selected regimen of 1 primary dose for further clinical development. Moreover, the kinetics of RSV-A and RSV-B neutralization titers are in line with observations made in a study with another RSV prefusion F protein–based vaccine [24]. That study did not assess CMI responses.

While protective RSV immune effector mechanisms have not yet been clearly defined, some evidence suggests that serum neutralization antibodies against the virus may be a functional correlate of immunity [9, 10, 25]. In older adults, RSV-specific serum neutralization titers are inversely correlated with risk of reinfection [11–13], disease severity [13], and hospitalization [25, 26]. Antibody functions beyond neutralization may also play a role in controlling RSV infections, as recently suggested by preclinical [27] and human [28] experimental RSV infection models. Conversely, RSV-specific T-cell responses are thought to play a role in controlling disease severity, and reductions in these responses due to immunosenescence likely contribute to the susceptibility of older adults to severe RSV disease [14, 15]. The humoral and cell-mediated immune responses in all age groups that persisted for at least 1 year postvaccination with a single dose of the RSVPreF3 OA vaccine described here are consistent with protection against RSV-related acute respiratory illness and severe lower respiratory tract disease during at least 1 RSV season, as observed in the phase 3 AReSVi-006 efficacy study [21]. Furthermore, the strong and consistent neutralization responses observed against RSV-A and RSV-B align with a recent study demonstrating that the RSVPreF3 OA vaccine effectively enhances neutralization responses against a range of antigenically distinct RSV variants [18].

The present study is the first of the RSVPreF3 OA program with immunogenicity results up to 1 year postvaccination after a single dose in individuals aged ≥60 years, including those ≥80 years. As the risk of medically attended RSV infections and hospitalizations increases with age [5, 29–32] due to immunosenescence [33, 34], this is a particularly vulnerable population. While all age groups mounted a marked increase in neutralization titers 1 month after vaccination, a trend toward lower RSV-A– and RSV-B–specific humoral immune responses was observed in participants aged ≥70 years as compared with those aged 60 to 69 years, which might be attributed to immunosenescence. Nevertheless, the 95% CIs were overlapping, and the responses converged back to similar levels across all age groups over time. No trend toward lower cell-mediated immune responses was observed by age. The results presented here thus suggest that similar protection and immune persistence might be achieved in the different age groups.

In this ongoing clinical study, we confirmed that a single dose of the vaccine was well tolerated and had an acceptable safety profile in older adults, similar to the interim reactogenicity and safety results of the phase 3 efficacy study [21]. One SAE—a case of Guillain-Barré syndrome, which eventually resolved—was considered by the investigator to be causally related to the vaccine. Despite a temporal relationship of this case with RSVPreF3 OA vaccination, there was insufficient evidence to confirm the diagnosis due to a lack of examination results excluding alternative etiologies. While this syndrome has been historically associated with other vaccines—such as the swine flu vaccine [35], the recombinant zoster vaccine [36], and some COVID-19 vaccines [37]—no other cases of Guillain-Barré syndrome have been reported in any other RSVPreF3 OA clinical studies as of August 2023.

A limitation of this study is that it was not designed to compare the results with a placebo control group. To overcome this limitation for the interpretation of the immune response, we compared the neutralization titers, IgG concentrations, and polypositive CD4+ T-cell frequencies at the different postvaccination time points with the prevaccination levels. We deem comparisons with prevaccination levels appropriate, as dropout rates were low and there was no selection bias caused by dropout; that is, the population had similar characteristics (eg, age) when the study started (day 1) and at month 12. Yet, safety data could be compared with the phase 3 efficacy study, which was performed in a similar setting in the same period [21].

In conclusion, a single dose of the RSVPreF3 OA vaccine elicited an RSV-A– and RSV-B–specific humoral and RSVPreF3-specific cell-mediated immune response that persisted up to 1 year postvaccination in adults aged ≥60 years. This in line with the high vaccine efficacy against RSV-related disease during at least 1 RSV season that was demonstrated in the AReSVi-006 phase 3 efficacy study. Moreover, we confirmed that the vaccine was well tolerated and had an acceptable safety profile. Future results of this trial will shed light on the longer-term immune persistence up to 3 years after 1-dose vaccination and on the most optimal revaccination strategies to offer longevity of immunologic protection. With the ongoing AReSVi-006 trial that is evaluating longer-term efficacy of RSVPreF3 OA up to 3 years postvaccination and the need for revaccination [21], these results will describe the longevity of protection and immunogenicity, as well as the long-term safety profile of RSVPreF3 OA in older adults.

## Supplementary Data

Supplementary materials are available at *The Journal of Infectious Diseases* online (http://jid.oxfordjournals.org/). Supplementary materials consist of data provided by the author that are published to benefit the reader. The posted materials are not copyedited. The contents of all supplementary data are the sole responsibility of the authors. Questions or messages regarding errors should be addressed to the author.

## Supplementary Material

jiad546_Supplementary_Data
