# Supplementary material for: Immunogenicity and Safety Following 1 Dose of AS01E-Adjuvanted Respiratory Syncytial Virus Prefusion F Protein Vaccine in Older Adults: A Phase 3 Trial
Source: J Infect Dis. 2023 Dec 14;230(1):e102–10. doi: 10.1093/infdis/jiad546 (PMC11272088; doi:10.1093/infdis/jiad546)
Supplement: jiad546_Supplementary_Data [file jiad546_supplementary_data.zip › Supplementary_methods.docx]

**Title:** Immunogenicity and safety following one dose of AS01_E_-adjuvanted respiratory syncytial virus prefusion F protein vaccine in older adults: a phase 3 trial

**Running title:** RSVPreF3 OA vaccine in older adults

**Authors and affiliations:**

- **Tino F Schwarz**, Institute of Laboratory Medicine and Vaccination Centre, Klinikum Würzburg Mitte, Campus Juliusspital, 97074 Würzburg, Germany
- **Shinn-Jang Hwang**, En Chu Kong Hospital, New Taipei City, Taipei Veterans General Hospital, and National Yang Ming Chiao Tung University School of Medicine, Taipei, 112, Taiwan
- **Pedro Ylisastigui**, Alliance for MultiSpecialty Research, Fort Myers, FL 33912, United States
- **Chiu-Shong Liu**, China Medical University and China Medical University Hospital, Taichung, 404327, Taiwan
- **Kenji Takazawa**, Medical Corporation Shinanokai, Shinanozaka Clinic, Tokyo, 160-0017, Japan
- **Makoto Yono**, Nishi-Kumamoto Hospital, Kumamoto, 861-4157, Japan
- **John E Ervin**, Alliance for Multispecialty Research, Kansas City, MO 64114, United States
- **Charles P Andrews**, IMA Research San Antonio, San Antonio, TX 78229, United States
- **Charles Fogarty**, Spartanburg Medical Research, Spartanburg, SC 29303, United States
- **Tamara Eckermann**, Praxis Dr. med. Irmgard Maier-Bosse, 80339 Munich, Germany
- **Delphine Collete**, GSK, 1330 Rixensart, Belgium
- **Magali de Heusch**, GSK, 1300 Wavre, Belgium
- **Nathalie De Schrevel**, GSK, 1330 Rixensart, Belgium
- **Bruno Salaun**, GSK, 1330 Rixensart, Belgium
- **Axel Lambert**, GSK, 1300 Wavre, Belgium
- **Céline Maréchal**, GSK, 1300 Wavre, Belgium
- **Aurélie Olivier**, GSK, 1300 Wavre, Belgium
- **Phoebe Nakanwagi**, GSK, 1300 Wavre, Belgium
- **Marc Lievens***, GSK, 1300 Wavre, Belgium
- **Veronica Hulstrøm**, GSK, 1300 Wavre, Belgium

*** Corresponding author:** Marc Lievens

Address: GSK, 1300 Wavre, Belgium

e-mail: [Marc.lievens@gsk.com](mailto:Marc.lievens@gsk.com)

Tel: +32 477 624485

**Supplementary methods**

***Inclusion and exclusion criteria***

**Inclusion criteria for enrollment**

All participants had to satisfy all of the following criteria at study entry:

- Male or female participants ≥60 years of age at first vaccination, who lived in the community or in a long-term care facility (LTCF).
- Participants who, in the opinion of the investigator, could and would comply with the requirements of the protocol (e.g., complete the diary cards, attend regular phone calls/study site visits, access and utilize a phone or other electronic communications).
  *Note: In case of physical incapacity that would preclude the self-completion of the diary cards, either site staff could assist the participant (for activities performed during site visits) or the participant could assign a caregiver to assist him/her with this activity (for activities performed at home or in the LTCF). However, at no time could the site staff or caregiver evaluate the participant’s health status while answering diaries, or make decisions on behalf of the participant.*
- Written or witnessed informed consent had to be obtained from the participant prior to performance of any study-specific procedure.
- Participants who were medically stable in the opinion of the investigator at the time of first vaccination. Patients with chronic stable medical conditions with or without specific treatment, such as diabetes, hypertension, or cardiac disease, were allowed to participate in this study if considered by the investigator as medically stable.

**Exclusion criteria for enrollment**

The potential participant could not be included in the study if any exclusion criterion applied:

- Medical conditions
- Any confirmed or suspected immunosuppressive or immunodeficient condition resulting from disease (e.g., current malignancy, human immunodeficiency virus) or immunosuppressive/cytotoxic therapy (e.g., medication used during cancer chemotherapy, organ transplantation, or to treat autoimmune disorders), based on medical history and physical examination (no laboratory testing required).
- History of any reaction or hypersensitivity likely to be exacerbated by any component of the study vaccine.
- Hypersensitivity to latex.
- Serious or unstable chronic illness.
- Recurrent or un-controlled neurological disorders or seizures. Participants with medically controlled active or chronic neurological diseases could be enrolled in the study as per investigator assessment, provided that their condition would allow them to comply with the requirements of the protocol (e.g., complete the diary cards, attend regular phone calls/study site visits).
- Significant underlying illness that in the opinion of the investigator would be expected to prevent completion of the study (e.g., life-threatening disease likely to limit survival to less than three years).
- Any medical condition that in the judgment of the investigator would make intramuscular injection unsafe.
- Any history of dementia or any medical condition that moderately or severely impaired cognition.
  *Note: If deemed necessary for clinical evaluation, the investigator could use tools such as Mini-Mental State Examination (MMSE), Mini-Cog, or Montreal Cognitive Assessment (MoCA) to determine cognition levels of the participant.*
- Prior/concomitant therapy
- Use of any investigational or non-registered product (drug, vaccine, or medical device) other than the study vaccine during the period beginning 30 days before the first dose of study vaccine, or planned use during the study period.
- Planned or actual administration of a vaccine not foreseen by the study protocol in the period starting 30 days before each dose and ending 30 days after each dose of study vaccine administration, with the exception of inactivated, split-virion, and subunit influenza vaccines, which could be administered up to 14 days before or from 14 days after each study dose.
  *Note: In case an emergency mass vaccination for an unforeseen public health threat (e.g., a pandemic) was recommended and/or organized by the public health authorities, outside the routine immunization program, the time period described above could be reduced if necessary for that vaccine (e.g., COVID-19 vaccines), provided it was used according to the local governmental recommendations and that the Sponsor was notified accordingly.*
- Previous vaccination with an RSV vaccine.
- Administration of long-acting immune-modifying drugs (e.g., infliximab) or planned administration of such drugs at any time during the study period.
- Administration of immunoglobulins and/or any blood products or plasma derivatives during the period starting 90 days before the first dose of study vaccine or planned administration during the study period.
- Chronic administration (defined as more than 14 consecutive days in total) of immunosuppressants or other immune-modifying drugs during the period starting 90 days prior to the first vaccine dose or planned administration during the study period. For corticosteroids, this meant prednisone ≥20 mg/day, or equivalent. Inhaled and topical steroids were allowed.
- Prior/concurrent clinical study experience
  - Concurrently participating in another clinical study, at any time during the study period, in which the participant has been or would be exposed to an investigational or a non-investigational vaccine/product (drug or invasive medical device).
- Other exclusions
  - History of chronic alcohol consumption and/or drug abuse as deemed by the investigator to render the potential participant unable/unlikely to provide accurate safety reports or comply with study procedures.
  - Bedridden participants.
  - Planned move during the study period that would have prohibited participation in the trial until the study end. This included:
- Planned move during the study period to another LTCF that would have prohibited participation in the trial until the study end.
- Planned move from the community to a LTCF that would have prohibited participation in the trial until the study end.
- Participation of any study personnel or their immediate dependents, family, or household members.

***Allocation of participants to immunogenicity subsets***

Two subsets were generated for the immunogenicity analyses, a humoral immunogenicity subset and a cell-mediated immunogenicity (CMI) subset. Allocation of participants to these immunogenicity subsets was performed using the SBIR system.

The target sample sizes of the subsets are detailed below:

| Subset | RSV_annual | RSV_flexible revaccination | RSV_1 dose |
| --- | --- | --- | --- |
| Humoral immunogenicity subset, N | ~345^a^ | All participants (~330) | All participants (~330) |
| CMI subset, N | ~345^a^ | ~115 | ~115 |

RSV, respiratory syncytial virus; N, aimed number of participants within each subset and study group; CMI, cell-mediated immunogenicity.

RSV_annual: group of participants receiving a first RSVPreF3 OA dose on day 1, followed by revaccination doses 12 months and 24 months post-dose 1.

RSV_flexible revaccination: group of participants receiving a first RSVPreF3 OA dose on day 1, and a revaccination dose 24 months post-dose 1.

RSV_1 dose: group of participants receiving a single dose on day 1.

^a^ For the RSV_annual group, the same ~345 participants are part of both the humoral immunogenicity and CMI subsets. The remaining ~645 participants do not have blood draws and are only followed up for safety and reactogenicity.

• **Humoral immunogenicity subset:** These participants have blood samples collected for testing of humoral immunity at each visit applicable for their study group.

• **CMI subset:** These participants have additional blood samples collected for CMI testing at each visit applicable for their study group.

***Study objectives***

| Objectives | Endpoints |
| --- | --- |
| Primary |  |
| To evaluate the humoral immune response following a 1-dose primary schedule of RSVPreF3 OA investigational vaccine up to 12 months post-dose 1. | Humoral immune response at pre-vaccination (day 1), 30 days post-dose 1 (day 31), and at 6 and 12 months post-dose 1 (months 6 and 12), in a subset of participants:  • Neutralization titers against RSV-A  • Neutralization titers against RSV-B |
| Secondary |  |
| To further evaluate the humoral immune response following a 1-dose primary schedule of RSVPreF3 OA investigational vaccine up to 12 months post-dose 1. | Humoral immune response at pre-vaccination (day 1), 30 days post-dose 1 (day 31), and at 6 and 12 months post-dose 1 (months 6 and 12), in a subset of participants:  • RSVPreF3-binding IgG antibody concentrations |
| To evaluate the humoral immune response following 1 dose of the RSVPreF3 OA investigational vaccine and following revaccination doses, up to study end. | Humoral immune response at months 18, 24, 30, and 36 post-dose 1, and at 1 month after each revaccination dose (months 13 and 25), in a subset of participants:  • Neutralization titers against RSV-A and RSV-B  • RSVPreF3-binding IgG antibody concentrations |
| To evaluate the CMI response following 1 dose of the RSVPreF3 OA investigational vaccine and following revaccination doses up to study end. | CMI response at pre-vaccination (day 1), 30 days post-dose 1 (day 31), at months 6, 12, 18, 24, 30, and 36 post-dose 1, and at 1 month after each revaccination dose (months 13 and 25), in a subset of participants:  • Frequency of RSVPreF3-specific CD4+ and/or CD8+ T cells expressing at least two activation markers including at least one cytokine among CD40L, 4-1BB, IL-2, TNF-α, IFN-γ, IL-13, IL-17 |
| To evaluate the safety and reactogenicity of each vaccination schedule of the RSVPreF3 OA investigational vaccine in all participants. | • Occurrence of solicited administration-site and systemic AEs during a 4-day follow-up period (i.e., on the day of vaccination and three subsequent days) after each dose.  • Occurrence of any unsolicited AE during a 30-day follow-up period (i.e., on the day of vaccination and 29 subsequent days) after each dose.  • Occurrence of all SAEs and pIMDs up to 6 months after each dose.  • Occurrence of fatal SAEs, SAEs related to the study vaccine, and pIMDs related to the study vaccine, from first dose (day 1) up to study end (month 36) |

RSVPreF3 OA, AS01_E_-adjuvanted respiratory syncytial virus prefusion F protein-based vaccine for older adults; RSV, respiratory syncytial virus; IgG, immunoglobulin G; CMI, cell-mediated immunogenicity; CD4/8/40L, cluster of differentiation 4/8/40 ligand; IL, interleukin; TNF-α, tumor necrosis factor alpha; IFN-γ, interferon gamma; AE, adverse event; SAE, serious adverse event; pIMD, potential immune-mediated disease.

***Solicited and unsolicited adverse events and grading scale***

The intensity of solicited adverse events (AEs) were assessed as described in the table below:

| Event | Grade | Parameter |
| --- | --- | --- |
| Administration-site solicited AE |  |  |
| Pain at administration site | 0 | None |
|  | 1 | Any pain neither interfering with nor preventing normal everyday activities |
|  | 2 | Painful when limb is moved and interferes with everyday activities |
|  | 3 | Significant pain at rest. Prevents normal everyday activities |
| Erythema at administration site | 0 | ≤20 mm |
|  | 1 | >20–≤50 mm |
|  | 2 | >50–≤100 mm |
|  | 3 | >100 mm |
| Swelling at administration site | 0 | ≤20 mm |
|  | 1 | >20–≤50 mm |
|  | 2 | >50–≤100 mm |
|  | 3 | >100mm |
| Systemic solicited AE |  |  |
| Fever | 0 | <38.0°C (100.4°F) |
|  | 1 | ≥38.0°C (100.4°F)–≤38.5°C (101.3°F) |
|  | 2 | >38.5°C (101.3°F)–≤39.0°C (102.2°F) |
|  | 3 | >39.0°C (102.2°F) |
| Headache, fatigue, myalgia, arthralgia | 0 | Normal |
|  | 1 | Symptom that is easily tolerated |
|  | 2 | Symptom that interferes with normal activity |
|  | 3 | Symptom that prevents normal activity |

AE, adverse event.

The intensity of unsolicited AEs was assessed by the investigator and assigned to one of the following categories, based on the investigator’s clinical judgment:

- **1 (mild) =** An AE which is easily tolerated by the participant, causing minimal discomfort, and not interfering with everyday activities.
- **2 (moderate) =** An AE which is sufficiently discomforting to interfere with normal everyday activities.
- **3 (severe) =** An AE which prevents normal, everyday activities. Such an AE would, for example, prevent attendance at work and would necessitate the administration of corrective therapy.
