# Supplementary material for: Immunogenicity and Safety Following 1 Dose of AS01E-Adjuvanted Respiratory Syncytial Virus Prefusion F Protein Vaccine in Older Adults: A Phase 3 Trial
Source: J Infect Dis. 2023 Dec 14;230(1):e102–10. doi: 10.1093/infdis/jiad546 (PMC11272088; doi:10.1093/infdis/jiad546)
Supplement: jiad546_Supplementary_Data [file jiad546_supplementary_data.zip › Supplementary_table_1.docx]

***Supplementary table 1. RSV-A and RSV-B neutralization titers, by age group (per-protocol set for humoral immunogenicity)***

| **Timepoint** | **GMT** | | **MGI** | |
| --- | --- | --- | --- | --- |
| **RSV-A neutralization titer** | **N** | **GMT (95% CI), ED60** | **N** | **MGI (95% CI)** |
| **60–69 years** |  |  |  |  |
| Day 1 | 478 | 849.2 (787.0–916.3) | - | - |
| Day 31 | 458 | 9862.6 (8992.8–10816.4) | 457 | 11.6 (10.6–12.6) |
| Month 6 | 448 | 3752.4 (3442.0–4090.7) | 447 | 4.5 (4.2–4.8) |
| Month 12 | 431 | 2785.5 (2541.5–3052.9) | 430 | 3.3 (3.1–3.5) |
| **70–79 years** |  |  |  |  |
| Day 1 | 379 | 852.6 (784.7–926.5) | - | - |
| Day 31 | 353 | 8486.9 (7578.2–9504.6) | 353 | 10.0 (8.9–11.1) |
| Month 6 | 352 | 3738.2 (3391.2– 4120.7) | 352 | 4.5 (4.1–4.9) |
| Month 12 | 327 | 2469.5 (2235.7–2727.7) | 327 | 2.9 (2.7–3.2) |
| **≥80 years** |  |  |  |  |
| Day 1 | 128 | 953.0 (829.6–1094.7) | - | - |
| Day 31 | 126 | 8234.3 (6855.5– 9890.3) | 126 | 8.8 (7.3–10.5) |
| Month 6 | 124 | 3768.1 (3214.9–4416.4) | 124 | 4.0 (3.4–4.6) |
| Month 12 | 112 | 2826.5 (2390.0–3342.7) | 112 | 2.9 (2.5–3.4) |
| **RSV-B neutralization titer** | **N** | **GMT (95% CI), ED60** | **N** | **MGI (95% CI)** |
| **60–69 years** |  |  |  |  |
| Day 1 | 479 | 1273.0 (1177.2–1376.7) | - | - |
| Day 31 | 458 | 10302.5 (9480.6–11195.6) | 458 | 8.1 (7.5–8.9) |
| Month 6 | 448 | 4500.6 (4168.5–4859.2) | 448 | 3.6 (3.3–3.8) |
| Month 12 | 431 | 2972.6 (2735.4– 3230.4) | 431 | 2.3 (2.2–2.5) |
| **70–79 years** |  |  |  |  |
| Day 1 | 379 | 1151.8 (1062.6–1248.5) | - | - |
| Day 31 | 353 | 9044.1 (8239.1–9927.9) | 353 | 7.8 (7.1–8.6) |
| Month 6 | 352 | 4156.1 (3803.6–4541.3) | 352 | 3.7 (3.4–4.0) |
| Month 12 | 327 | 2822.2 (2570.3–3098.8) | 327 | 2.4 (2.2–2.6) |
| **≥80 years** |  |  |  |  |
| Day 1 | 128 | 1355.1 (1160.3–1582.6) | - | - |
| Day 31 | 126 | 8962.2 (7610.1–10554.7) | 126 | 6.7 (5.7–7.8) |
| Month 6 | 124 | 3986.8 (3424.1– 4642.0) | 124 | 3.0 (2.6–3.4) |
| Month 12 | 112 | 2750.3 (2344.0–3227.0) | 112 | 2.1 (1.8–2.5) |

RSV, respiratory syncytial virus; GMT, geometric mean neutralization titer; MGI, mean geometric increase at given timepoint over baseline (day 1); N, number of participants with available results; CI, confidence interval; ED60, estimated dilution 60.
