# Supplementary material for: Immunogenicity and Safety Following 1 Dose of AS01E-Adjuvanted Respiratory Syncytial Virus Prefusion F Protein Vaccine in Older Adults: A Phase 3 Trial
Source: J Infect Dis. 2023 Dec 14;230(1):e102–10. doi: 10.1093/infdis/jiad546 (PMC11272088; doi:10.1093/infdis/jiad546)
Supplement: jiad546_Supplementary_Data [file jiad546_supplementary_data.zip › Supplementary_table_2.docx]

***Supplementary table 2. RSV-A and RSV-B neutralization titers, by region (per-protocol set for humoral immunogenicity)***

| **Timepoint** | **GMT** | | **MGI** | |
| --- | --- | --- | --- | --- |
| **RSV-A neutralization titer** | **N** | **GMT (95% CI), ED60** | **N** | **MGI (95% CI)** |
| **North America** |  |  |  |  |
| Day 1 | 230 | 958.5 (853.8–1076.1) | - | - |
| Day 31 | 214 | 11236.0 (9808.0–12871.8) | 214 | 11.4 (9.9–13.1) |
| Month 6 | 213 | 4395.0 (3878.4–4980.3) | 213 | 4.7 (4.2–5.2) |
| Month 12 | 194 | 3000.6 (2623.4–3432.2) | 194 | 3.1 (2.8–3.5) |
| **Europe** |  |  |  |  |
| Day 1 | 409 | 927.0 (857.2–1002.5) | - | - |
| Day 31 | 384 | 9512.3 (8567.6–10561.2) | 383 | 10.3 (9.3–11.4) |
| Month 6 | 373 | 3956.7 (3590.7–4359.9) | 372 | 4.3 (4.0–4.7) |
| Month 12 | 360 | 2829.5 (2559.3–3128.2) | 359 | 3.0 (2.8–3.3) |
| **Asia** |  |  |  |  |
| Day 1 | 346 | 740.4 (680.5–805.6) | - | - |
| Day 31 | 339 | 7567.9 (6777.7–8450.2) | 339 | 10.3 (9.2–11.4) |
| Month 6 | 338 | 3195.8 (2912.9–3506.2) | 338 | 4.3 (4.0–4.7) |
| Month 12 | 316 | 2319.8 (2103.0–2558.8) | 316 | 3.2 (2.9–3.5) |
| **RSV-B neutralization titer** | **N** | **GMT (95% CI), ED60** | **N** | **MGI (95% CI)** |
| **North America** |  |  |  |  |
| Day 1 | 230 | 1242.2 (1102.2–1400.0) | - | - |
| Day 31 | 214 | 11351.8 (10098.1–12761.1) | 214 | 8.9 (7.8–10.2) |
| Month 6 | 213 | 4612.6 (4116.7–5168.3) | 213 | 3.8 (3.4–4.2) |
| Month 12 | 194 | 3209.2 (2806.8–3669.3) | 194 | 2.5 (2.2–2.8) |
| **Europe** |  |  |  |  |
| Day 1 | 410 | 1254.3 (1152.5–1365.1) | - | - |
| Day 31 | 384 | 9738.9 (8879.9–10681.0) | 384 | 7.9 (7.2–8.7) |
| Month 6 | 373 | 4243.5 (3882.5–4638.0) | 373 | 3.4 (3.2–3.7) |
| Month 12 | 360 | 2673.2 (2440.8–2927.8) | 360 | 2.1 (2.0–2.3) |
| **Asia** |  |  |  |  |
| Day 1 | 346 | 1207.7 (1113.7–1309.5) | - | - |
| Day 31 | 339 | 8562.9 (7783.2–9420.7) | 339 | 7.1 (6.4–7.8) |
| Month 6 | 338 | 4163.1 (3824.0–4532.2) | 338 | 3.5 (3.2–3.7) |
| Month 12 | 316 | 2950.8 (2701.4–3223.4) | 316 | 2.5 (2.3–2.6) |

RSV, respiratory syncytial virus; GMT, geometric mean neutralization titer; MGI, mean geometric increase at given timepoint over baseline (day 1); N, number of participants with available results; CI, confidence interval; ED60, estimated dilution 60.
