# Supplementary material for: Immunogenicity and Safety Following 1 Dose of AS01E-Adjuvanted Respiratory Syncytial Virus Prefusion F Protein Vaccine in Older Adults: A Phase 3 Trial
Source: J Infect Dis. 2023 Dec 14;230(1):e102–10. doi: 10.1093/infdis/jiad546 (PMC11272088; doi:10.1093/infdis/jiad546)
Supplement: jiad546_Supplementary_Data [file jiad546_supplementary_data.zip › Supplementary_table_3.docx]

***Supplementary table 3. Frequency of RSVPreF3-specific polypositive CD4+ T cells expressing at least two activation markers including at least one cytokine among CD40L, 4-1BB, IL-2, TNF-α, IFN-ɣ, IL-13, and IL-17, by age group (per-protocol set for cell-mediated immunogenicity)***

| Age category  Time point | N | Median (Q1–Q3) |
| --- | --- | --- |
| 60–69 years |  |  |
| Day 1 | 232 | 194.5 (71.5–385.0) |
| Day 31 | 200 | 1351.0 (882.0–2026.5) |
| Month 6 | 215 | 741.0 (462.0–1049.0) |
| Month 12 | 217 | 582.0 (388.0–927.0) |
| 70–79 years |  |  |
| Day 1 | 183 | 182.0 (70.0–334.0) |
| Day 31 | 156 | 1291.5 (788.5–2192.5) |
| Month 6 | 169 | 645.0 (417.0–1041.0) |
| Month 12 | 163 | 534.0 (328.0–898.0) |
| ≥80 years |  |  |
| Day 1 | 56 | 189.0 (62.5–380.0) |
| Day 31 | 52 | 1413.0 (776.5–2354.5) |
| Month 6 | 52 | 547.5 (340.5–1187.5) |
| Month 12 | 58 | 600.5 (280.0–1054.0) |

RSVPreF3, respiratory syncytial virus prefusion F protein; CD4/CD40L, cluster of differentiation 4/40 ligand; CD40L, CD40 ligand; IL, interleukin; TNF-α, tumor necrosis factor alpha; IFN-γ, interferon gamma; CMI, cell-mediated immunogenicity; N, number of participants with available results; Q1, 25^th^ percentile; Q3, 75^th^ percentile.
