# Supplementary material for: Immunogenicity and Safety Following 1 Dose of AS01E-Adjuvanted Respiratory Syncytial Virus Prefusion F Protein Vaccine in Older Adults: A Phase 3 Trial
Source: J Infect Dis. 2023 Dec 14;230(1):e102–10. doi: 10.1093/infdis/jiad546 (PMC11272088; doi:10.1093/infdis/jiad546)
Supplement: jiad546_Supplementary_Data [file jiad546_supplementary_data.zip › Supplementary_table_4.docx]

***Supplementary table 4. Summary of all reported serious adverse events with onset within 6 months following vaccination, by MedDRA Primary System Organ Class and High Level Term (exposed set)***

| Primary System Organ Class | N=1653  n | % (95% CI) |
| --- | --- | --- |
| Preferred Term |  |  |
| Any SAE | 65 | 3.9 (3.0–5.0) |
| Cardiac disorders | 14 | 0.8 (0.5–1.4) |
| *Atrial fibrillation* | *5* | *0.3 (0.1–0.7)* |
| *Sinus arrest* | *1* | *0.1 (0.0–0.3)* |
| *Coronary artery disease* | *4* | *0.2 (0.1–0.6)* |
| *Myocardial infarction* | *2* | *0.1 (0.0–0.4)* |
| *Acute myocardial infarction* | *1* | *0.1 (0.0–0.3)* |
| *Angina pectoris* | *1* | *0.1 (0.0–0.3)* |
| *Cardiac failure congestive* | *1* | *0.1 (0.0–0.3)* |
| Neoplasms benign, malignant and unspecified (incl cysts and polyps) | 12 | 0.7 (0.4–1.3) |
| *Breast cancer* | *2* | *0.1 (0.0–0.4)* |
| *Lung adenocarcinoma* | *2* | *0.1 (0.0–0.4)* |
| *Small cell lung cancer* | *2* | *0.1 (0.0–0.4)* |
| *Rectal cancer* | *1* | *0.1 (0.0–0.3)* |
| *Lymphoma* | *1* | *0.1 (0.0–0.3)* |
| *Squamous cell carcinoma* | *1* | *0.1 (0.0–0.3)* |
| *Malignant pleural effusion* | *1* | *0.1 (0.0–0.3)* |
| *Pancreatic carcinoma* | *1* | *0.1 (0.0–0.3)* |
| *Prostate cancer* | *1* | *0.1 (0.0–0.3)* |
| Injury, poisoning and procedural complications | 11 | 0.7 (0.3–1.2) |
| *Clavicle fracture* | *1* | *0.1 (0.0–0.3)* |
| *Femoral neck fracture* | *1* | *0.1 (0.0–0.3)* |
| *Fibula fracture* | *1* | *0.1 (0.0–0.3)* |
| *Hip fracture* | *1* | *0.1 (0.0–0.3)* |
| *Radius fracture* | *1* | *0.1 (0.0–0.3)* |
| *Joint dislocation* | *1* | *0.1 (0.0–0.3)* |
| *Multiple fractures* | *1* | *0.1 (0.0–0.3)* |
| *Procedural pneumothorax* | *1* | *0.1 (0.0–0.3)* |
| *Subdural hematoma* | *1* | *0.1 (0.0–0.3)* |
| *Wound dehiscence* | *1* | *0.1 (0.0–0.3)* |
| *Craniofacial injury* | *1* | *0.1 (0.0–0.3)* |
| *Spinal fracture* | *1* | *0.1 (0.0–0.3)* |
| *Thermal burn* | *1* | *0.1 (0.0–0.3)* |
| *Rib fracture* | *1* | *0.1 (0.0–0.3)* |
| Infections and infestations | 10 | 0.6 (0.3–1.1) |
| *Cellulitis* | *2* | *0.1 (0.0–0.4)* |
| *COVID-19* | *1* | *0.1 (0.0–0.3)* |
| *COVID-19 pneumonia* | *1* | *0.1 (0.0–0.3)* |
| *Urinary tract infection* | *2* | *0.1 (0.0–0.4)* |
| *Colonic abscess* | *1* | *0.1 (0.0–0.3)* |
| *Helicobacter gastritis* | *1* | *0.1 (0.0–0.3)* |
| *Post procedural infection* | *1* | *0.1 (0.0–0.3)* |
| *Gastroenteritis viral* | *1* | *0.1 (0.0–0.3)* |
| Nervous system disorders | 8 | 0.5 (0.2–1.0) |
| *Syncope* | *2* | *0.1 (0.0–0.4)* |
| *Loss of consciousness* | *1* | *0.1 (0.0–0.3)* |
| *Guillain-Barré syndrome* | *1* | *0.1 (0.0–0.3)* |
| *Cerebrovascular accident* | *1* | *0.1 (0.0–0.3)* |
| *Polyneuropathy in malignant disease* | *1* | *0.1 (0.0–0.3)* |
| *Cerebrospinal fluid leakage* | *1* | *0.1 (0.0–0.3)* |
| *Parkinson's disease* | *1* | *0.1 (0.0–0.3)* |
| Musculoskeletal and connective tissue disorders | 6 | 0.4 (0.1–0.8) |
| *Chondrolysis* | *1* | *0.1 (0.0–0.3)* |
| *Osteochondrosis* | *1* | *0.1 (0.0–0.3)* |
| *Osteoarthritis* | *1* | *0.1 (0.0–0.3)* |
| *Spinal osteoarthritis* | *1* | *0.1 (0.0–0.3)* |
| *Intervertebral disc protrusion* | *1* | *0.1 (0.0–0.3)* |
| *Sacroiliac joint dysfunction* | *1* | *0.1 (0.0–0.3)* |
| *Spinal stenosis* | *1* | *0.1 (0.0–0.3)* |
| Gastrointestinal disorders | 4 | 0.2 (0.1–0.6) |
| *Abdominal wall hematoma* | *1* | *0.1 (0.0–0.3)* |
| *Small intestinal obstruction* | *1* | *0.1 (0.0–0.3)* |
| *Gastric ulcer* | *1* | *0.1 (0.0–0.3)* |
| *Diabetic gastroparesis* | *1* | *0.1 (0.0–0.3)* |
| General disorders and administration-site conditions | 3 | 0.2 (0.0–0.5) |
| *Death* | *2* | *0.1 (0.0–0.4)* |
| *Pyrexia* | *1* | *0.1 (0.0–0.3)* |
| Respiratory, thoracic and mediastinal disorders | 3 | 0.2 (0.0–0.5) |
| *Sleep apnea syndrome* | *1* | *0.1 (0.0–0.3)* |
| *Chronic obstructive pulmonary disease* | *1* | *0.1 (0.0–0.3)* |
| *Pneumothorax* | *1* | *0.1 (0.0–0.3)* |
| Vascular disorders | 3 | 0.2 (0.0–0.5) |
| *Hypertensive crisis* | *1* | *0.1 (0.0–0.3)* |
| *Thrombosis* | *1* | *0.1 (0.0–0.3)* |
| *Arteriosclerosis* | *1* | *0.1 (0.0–0.3)* |
| Blood and lymphatic system disorders | 2 | 0.1 (0.0–0.4) |
| *Anemia* | *1* | *0.1 (0.0–0.3)* |
| *Nephrogenic anemia* | *1* | *0.1 (0.0–0.3)* |
| *Pancytopenia* | *1* | *0.1 (0.0–0.3)* |
| Hepatobiliary disorders | 2 | 0.1 (0.0–0.4) |
| *Cholelithiasis* | *2* | *0.1 (0.0–0.4)* |
| Investigations | 1 | 0.1 (0.0–0.3) |
| *International normalized ratio increased* | *1* | *0.1 (0.0–0.3)* |
| Metabolism and nutrition disorders | 1 | 0.1 (0.0–0.3) |
| *Hypokalemia* | *1* | *0.1 (0.0–0.3)* |
| Product issues | 1 | 0.1 (0.0–0.3) |
| *Device malfunction* | *1* | *0.1 (0.0–0.3)* |
| Reproductive system and breast disorders | 1 | 0.1 (0.0–0.3) |
| *Ovarian cyst* | *1* | *0.1 (0.0–0.3)* |
| Skin and subcutaneous tissue disorders | 1 | 0.1 (0.0–0.3) |
| *Dermal cyst* | *1* | *0.1 (0.0–0.3)* |

MedDRA, Medical Dictionary for Regulatory Activities; N, number of participants; n/% number/percentage of participants presenting at least one type of SAE; CI, confidence interval; SAE, serious adverse event.
